# Intraoperative Suction-Assisted Kyphoplasty for Immediate Removal of Posterior PMMA Extravasation in Decompressed Vertebral Compression Fractures

**DOI:** 10.3390/jcm15020481

**Published:** 2026-01-07

**Authors:** Yu-Chuan Tsuei, Hsin-Tzu Lu, Yung-Fu Hsu, Shih-Hao Cheng, William Chu, Woei-Chyn Chu

**Affiliations:** 1 Department of Biomedical Engineering, National Yang-Ming Chiao-Tung University, Taipei 11221, Taiwan; dontspider.be06@nycu.edu.tw (Y.-C.T.);; 2 Department of Orthopedics, Cheng Hsin General Hospital, Taipei 11220, Taiwan; 3 School of Nursing, National Taipei University of Nursing and Health Sciences, Taipei 112303, Taiwan

**Keywords:** osteoporotic vertebral compression fracture, cement leakage, percutaneous vertebroplasty, intravertebral decompression

## Abstract

**Background:** Vertebral compression fractures are prevalent in elderly patients with osteoporosis. While the vertebral augmentation procedure is often used for symptom control and improving life quality, cement leakage is still a significant complication of vertebral augmentation procedures. This case report describes a 92-year-old man with a T8 compression fracture who underwent kyphoplasty with low-viscosity bone cement after failed conservative treatment. **Methods:** A previously developed decompressed kyphoplasty technique using dual portals was employed to reduce intravertebral pressure; however, fluoroscopy revealed posterior leakage toward the spinal canal. A case-specific intraoperative modification of this established technique was then applied, converting the injection portal into a suction channel to aspirate extravasated cement before it hardened. **Results:** This approach averted spinal cord compromise, obviated the need for additional decompression surgery, and preserved neurological function. The patient achieved rapid pain relief and early mobilization. **Conclusions:** This case demonstrates how a suction-assisted intraoperative maneuver may be used to manage posterior cement leakage during decompressed kyphoplasty.

## 1. Introduction

Vertebral compression fractures (VCFs) are a frequent clinical challenge in older adults, particularly those with osteoporosis or other degenerative skeletal disorders [1]. Recent epidemiological data indicate prevalence rates of radiographic VCFs from 7.3% to 27% in community-dwelling elderly worldwide, with studies showing 7.3% prevalence in men and 11.1% in women among European older adults over age 70 [2]. Even minor traumas, such as a low-impact fall, may result in vertebral collapse, especially in osteoporotic bone with reduced density, as these fractures often occur at the thoracolumbar junction due to biomechanical vulnerability. The consequent pain and spinal deformity can significantly impair mobility, functional independence, and overall quality of life, leading to poorer physical performance like slower walking speeds and reduced functional reach. Without prompt treatment, VCFs often progress to chronic pain and are associated with increased comorbidity, including cardiovascular and pulmonary complications driven by reduced physical activity [3,4]. Kyphotic deformity from VCFs further compromises pulmonary function, with each fracture linked to up to a 9% reduction in forced vital capacity, contributing to restrictive lung disease patterns [5].

In contrast to traditional open surgical intervention with screw fixation to stabilize the fracture and correct kyphosis deformity, which may be risky for elderly patients, minimally invasive procedures—vertebroplasty and kyphoplasty—have transformed the management of VCFs [6,7,8]. These techniques are particularly effective for painful osteoporotic fractures, offering quick stabilization superior to conservative management in select cases. Recent meta-analyses show both procedures provide significant pain relief and functional improvement over conservative approaches like bracing and analgesics, with kyphoplasty demonstrating sustained benefits up to 12 months [9]. In vertebroplasty, polymethylmethacrylate (PMMA) bone cement is injected directly into the vertebral body to stabilize the fracture and provide rapid pain relief. Kyphoplasty extends this technique by first inflating a balloon to restore the vertebral body height and create an intravertebral cavity within the collapsed vertebra, allowing subsequent cement injection that may correct local kyphosis and limit extravasation [6,10]. The balloon tamp reduces injection pressure and creates a controlled space, lowering cement leakage compared to direct vertebroplasty injection. Studies confirm kyphoplasty improves vertebral compression rate and Cobb angle more effectively than vertebroplasty due to this cavity creation [11]. Nonetheless, cement leakage remains an ever-present concern—particularly posterior leakage toward the spinal canal—where it can compress neural elements and necessitate surgical decompression.

Cement leakage occurs when PMMA—intended to stabilize the vertebra—finds pathways into adjacent tissues, veins, or the spinal canal. This phenomenon arises due to the potential migration of the cement through fracture lines, venous channels, or cortical breaches during percutaneous vertebroplasty or kyphoplasty procedures. In clinical practice, PMMA cement is typically injected during its working phase, when it reaches a viscous, toothpaste-like consistency that allows controlled delivery. Nevertheless, the risk of cement extravasation remains substantial, with reported rates of 17–36% [12,13,14]. Leakage may exploit preexisting defects or weaknesses in vertebral cortical walls (especially posterior-wall disruption), traverse basivertebral or segmental veins, enter intervertebral discs, or follow needle tracts created during puncture [15]. Low cement viscosity at the time of injection, excessive cement volume, multilevel augmentation, or the presence of intravertebral clefts can all increase the likelihood of extravasation. Although injection may be discontinued at the first sign of leakage, most events are paravertebral and asymptomatic. By contrast, posterior leakage into the spinal canal or epidural space can result in spinal cord compression, leading to acute neurological deficits, paresthesia or even paraplegia [16,17,18,19]. Such scenarios often necessitate reoperation, a risk that is particularly concerning in elderly or frail patients, and the neurologic injury is usually permanent.

In our prior work, we introduced a “decompressed vertebroplasty and kyphoplasty” technique employing two portals—one was used for injecting low-viscosity cement and the other for venting served as a venting outlet for decompression of the vertebral body [20,21,22]. This dual-portal approach allows intravertebral pressure, air and fluid to be released while controlling aspiration flow. In that series, the leakage rate was only <6% [20], confined to the disc or paravertebral cortical defect regions. This previous work established decompressed kyphoplasty as a pressure-reducing method designed to enhance the safety of cement injection. Building on this foundation, the present case report describes a case-specific intraoperative modification to the decompressed kyphoplasty technique. Unlike the original method—which focuses on controlled pressure reduction during cement injection—the modification converts the injection portal into an active suction channel immediately upon detection of posterior cement extravasation. By preventing the cement from hardening near the spinal canal, this maneuver may help clinicians avoid neurologic injury and the risks of secondary surgery.

## 2. Materials and Methods

### 2.1. Patient History and Evaluation

**Figure 1:**
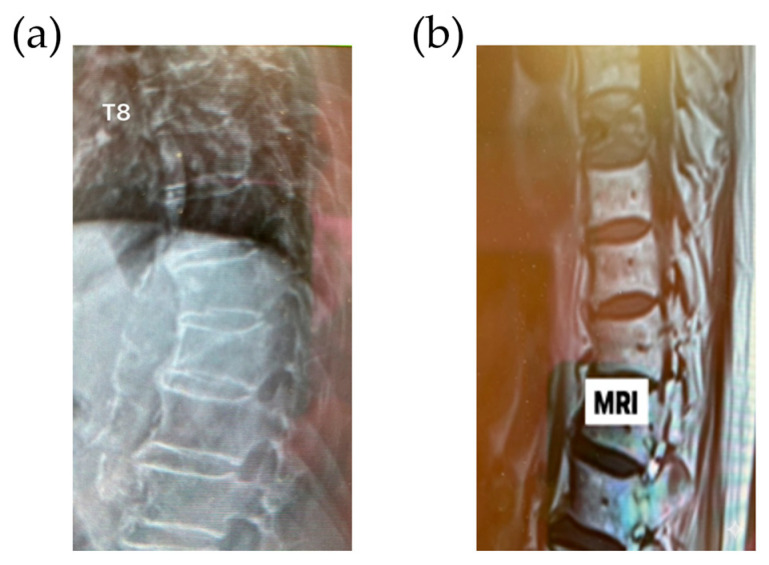
(**a**) Spine radiograph and (**b**) T1-weighted sagittal MRI demonstrating a compression fracture at T8 with partial loss of vertebral body height.

A 92-year-old man arrived with severe upper thoracic back pain that had persisted for one month following a minor fall accident at home. Initial conservative treatments, including pain medications, spinal bracing, and physical therapy, provided limited relief. Ongoing severe pain significantly impaired his daily function, prompting a surgical consultation. On clinical examination, localized tenderness was noted over the T8 vertebra. Movements like trunk rotation and forward flexion exacerbated the pain. Neurological evaluation showed no definite motor or sensory deficits in the lower extremities; however, the patient reported generalized weakness attributed to restricted mobility and persistent discomfort. Radiographs and MRI of the thoracic spine confirmed a new onset compression fracture at T8 with partial vertebral body collapse, as demonstrated in Figure 1. No acute spinal cord compression or canal compromise was observed; however, the patient’s advanced age and osteoporotic bone quality indicated a high risk of further collapse and potential neurological complications.

Given the limited response to conservative therapy, kyphoplasty was planned to stabilize the fracture and potentially restore vertebral height to correct kyphosis deformity.

### 2.2. Surgical Technique

**Figure 2:**
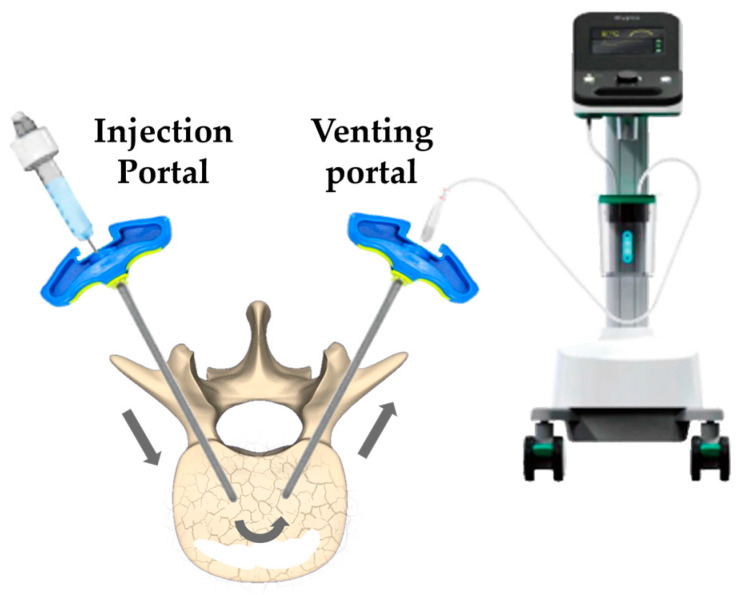
Schematic diagram of the decompressed percutaneous kyphoplasty procedure. A. Venting portal connected to a vacuum suction pump applying negative pressure. B. Injection portal connected to a syringe for cement delivery. Arrows indicate the direction of cement flow within the vertebral body.

Under general anesthesia, the patient was positioned prone on a radiolucent operating table, permitting continuous real-time fluoroscopic monitoring using a C-arm fluoroscopy unit in both anteroposterior and lateral projections. At the level of T8, two trocar cannulas (Medtronic, Minneapolis, MN, USA) were inserted transpedicularly into the vertebral body under fluoroscopic guidance (as illustrated in Figure 2):

1. Left pedicle (Injection Portal): Primarily used for cement injection, aided by differential pressure to facilitate controlled delivery.
2. Right pedicle (Venting Portal): Connected to continuous aspiration via a vacuum pump (DF-650, Doctor’s Friend Medical Instrument, Taichung, Taiwan) during the initial filling phase.

#### 2.2.1. Creation of a Vertebral Cavity

**Figure 3:**
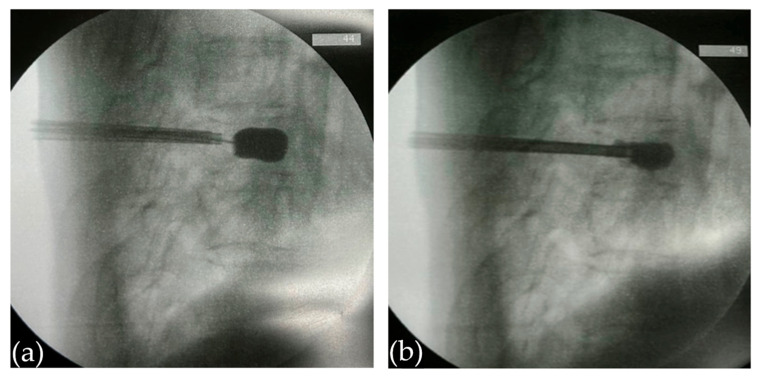
Lateral fluoroscopic views during kyphoplasty. (**a**) Creation of a vertebral cavity using an inflatable balloon tamp. (**b**) Monitoring of bone cement injection.

Following standard kyphoplasty procedures, an inflatable balloon tamp Xpander II inflatable bone tamps (Medtronic, Minneapolis, MN, USA) was advanced through each portal once correct positioning was confirmed under fluoroscopy. Gradual balloon inflation within the T8 vertebra elevated the collapsed endplates and compacted the cancellous bone, creating a cavity for cement placement (Figure 3a). After achieving partial restoration of vertebral height, the balloons were deflated and withdrawn. Subsequently, lavage and aspiration maneuvers were performed through both portals to verify communication between the channels and to help control the anticipated flow of cement.

#### 2.2.2. Cement Preparation and Initial Injection

PMMA bone cement (Howmedica Simplex P, Mahwah, NJ, USA) was prepared to a relatively low-viscosity, semi-liquid state (before the phase of “tooth-paste like”) to enable controlled injection while remaining sufficiently fluid for potential suction. Injection was commenced through the right portal, whereas the left portal was connected to continuous vacuum pressure at 560 mmHg. This level of suction force was found to be sufficient to mobilize low-viscosity cement without causing excessive removal of cancellous bone. Real-time fluoroscopic monitoring in both anteroposterior and lateral views was used to promptly identify any irregularities in cement spread (Figure 3b).

## 3. Results

### 3.1. Surveillance of Cement Leakage

**Figure 4:**
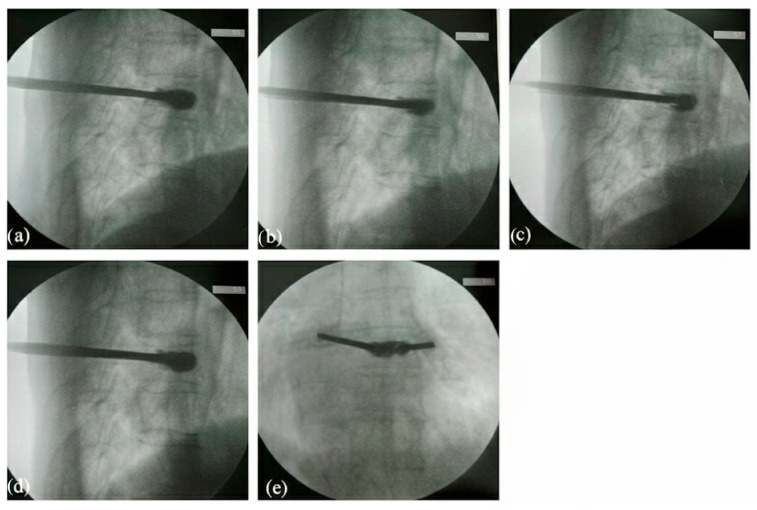
Fluoroscopic views during the decompressed kyphoplasty. (**a**) Observation of posteriorly cement extravasation. (**b**,**c**) Successful aspiration of leaked cement by suction connected to injection portal. (**d**,**e**) Final lateral and anteroposterior views following the decompression procedure.

After a period of injection and fulfilling of the vertebral body was achieved, lateral fluoroscopy revealed a small amount of PMMA extravasating posteriorly toward the spinal canal (Figure 4a). Recognizing the immediate risk of cord compression, the injection was immediately discontinued.

### 3.2. Decompression Maneuver

Although regular decompressed kyphoplasty was performed and contralateral continuous negative pressure was maintained during cement injection, posterior migration of cement was observed. At this point, the cement remained in a relatively low-viscosity, semi-liquid state, retaining both plasticity and fluidity. The injection portal was promptly converted into a suction channel, and by carefully advancing the suction tip through the cannula, the surgeon successfully aspirated the migrated cement from the posterior vertebral margin (Figure 4b,c). This maneuver not only removed the excessive cement but also prevented its polymerization adjacent to the spinal canal, thereby averting potential compression or permanent neurological injury. Importantly, the bulk of cement within the vertebral body remained intact, providing effective internal stabilization without the need for secondary decompression procedures, such as laminectomy (Figure 4d,e).

### 3.3. Postoperative Clinical and Radiographic Outcomes

No new neurological deficits were observed following the procedure. Motor and sensory examinations of the lower extremities remained unchanged compared with the preoperative baseline. Within 24 h, the patient experienced substantial pain reduction and was able to initiate early mobilization with a thoracolumbar orthosis. By postoperative day 2, he was able to ambulate short distances with assistance, and no delayed neurological symptoms, radicular pain, or signs of spinal canal compromise developed during hospitalization.

**Figure 5:**
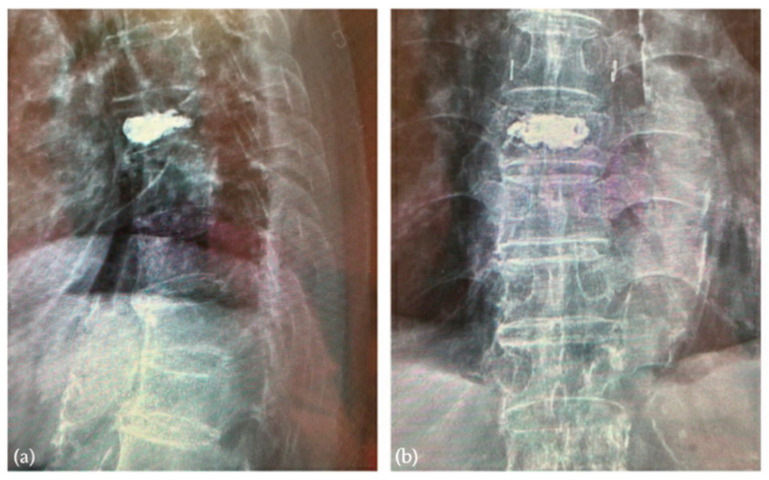
Postoperative radiographs showing no evidence of cement leakage. (**a**) Lateral view. (**b**) Anteroposterior view.

Postoperative radiographs confirmed stable intravertebral cement distribution without residual posterior extravasation, and the restored vertebral height achieved by balloon inflation was maintained (Figure 5). Because the patient exhibited no postoperative complications or new symptoms, additional CT imaging was not performed. Follow-up at two weeks showed no clinical deterioration, and radiographs demonstrated no cement migration, fragmentation, or new leakage, supporting the stability of the injected cement and the overall success of the decompressed kyphoplasty procedure.

## 4. Discussion

Osteoporosis poses a significant global public health challenge, with vertebral compression fractures representing a major source of chronic pain, disability, and diminished quality of life, and recent global burden analyses indicate rising incidence and years lived with disability for vertebral fractures in parallel with population aging and increased life expectancy worldwide [2,23]. Vertebroplasty and kyphoplasty are widely employed to address these issues by injecting PMMA cement into fractured vertebrae, thereby restoring structural integrity and alleviating pain, and contemporary clinical studies and systematic reviews confirm that, in carefully selected patients with osteoporotic vertebral compression fractures refractory to conservative therapy, these minimally invasive procedures can achieve more rapid pain relief and earlier functional recovery than non-operative management [24]. Although these techniques often provide rapid pain relief and functional improvement, complication rates—particularly cement leakage—remain a critical concern.

Cement leakage is typically attributed to pathological anatomical factors, such as fracture severity, cortical bone disruption or deficiency, posterior wall compromise, and the presence of intravertebral vacuum clefts [13,25,26], and recent clinical and imaging studies further emphasize that complex fracture morphology, disrupted endplates, intravertebral cleft–basivertebral foramen continuity, and defects of the anterior or lateral cortex substantially increase the likelihood of unintended cement escape from the vertebral body [27,28]. These conditions may lead to paravertebral/cortical, epidural or spinal canal, and intradiscal leakage, respectively. In addition, procedural factors also play a significant role, including excessive cement volume, overly rapid or high-pressure injection, and the resulting dispersion pattern [13,26,29].

Uniform cement distribution is generally considered essential for preventing recurrent vertebral collapse. In contrast, heterogeneous cement distribution may result in uneven stress transfer, predisposing the vertebra to recompression under external loads. Potential causes include unilateral filling, which increases asymmetric load transfer [30,31], or blocky aggregation rather than spongy, diffuse distribution, thereby failing to adequately span the vertebral endplates [32]. In clinical practice, maximizing cement filling within a collapsed vertebra can enhance distribution, restore vertebral height, and correct kyphotic angle [33]. However, excessive injection volume may increase the risk of cement leakage, potentially leading to severe complications such as adjacent vertebral fractures or pulmonary embolism [18].

Unlike conventional practice in which injection is performed during the “toothpaste-like” phase [34,35], the present case employed cement injection at a relatively low-viscosity, semi-liquid state. Previous studies have reported that this approach does not increase the risk of cement leakage, and have proposed adjunctive strategies such as stepwise cannula withdrawal during injection or repeat puncture for secondary injection [36,37]. In these reports, early cement injection was initiated at 2.0 min after mixing for Men-dec Spine (Tecres Medical, Verona, Italy) and at 2 min 5 s for OSTEOPAL V (Heraeus Medical GmbH, Wehrheim, Germany). In our study, the bone cement (Howmedica Simplex P, Mahwah, NJ, USA) was mixed for approximately 40 s, and the injection was prepared to begin at 3 min. In addition, decompressed kyphoplasty was performed, incorporating bilateral punctures for lavage and aspiration prior to cement delivery [38,39]. This technique has been shown to reduce intravertebral pressure and limit the risk of bone marrow, fat, or thrombus migration into the circulation. During injection, the venting portal was connected to vacuum suction to establish a pressure gradient, providing an outlet for excess cement or air. This allowed the surgeon to more reliably regulate intravertebral pressure and direct cement flow from the injection side to the contralateral side, thereby reducing leakage risk and promoting uniform distribution [20].

Existing strategies to reduce cement leakage in vertebral augmentation procedures are largely prevention-oriented and remain widely used in current clinical practice. These include the use of ultra-high-viscosity cement, high-pressure injection systems, meticulous biplanar fluoroscopic monitoring, and early termination of injection when posterior cement movement is suspected [40,41]. Collectively, these approaches aim to reduce the overall incidence of cement leakage through preemptive risk control and play an important role in routine vertebral augmentation procedures. However, when cement migration has already occurred intraoperatively—particularly in the presence of patient-specific anatomical or pathological factors—the immediate management options may be limited. It should be noted that cement leakage occurring posterior to the vertebral body posterior wall, especially when extending toward the spinal canal, represents a minority of leakage patterns reported in vertebral augmentation procedures and is not a frequent occurrence [18]. In the present case, posterior cement flow may have been influenced by the T8 compression fracture, as the vertebral body at this level is smaller than at more common fracture sites such as T11–L1. In such situations, precise cement volume control is critical: insufficient filling may compromise structural support, whereas excessive injection increases the risk of leakage or adjacent-level fractures.

In this case, bilateral punctures combined with kyphoplasty were performed to optimize vertebral filling. It should be noted that the use of relatively low-viscosity cement, as well as the intraoperative adaptation of temporarily converting the injection portal into a suction channel, are not explicitly described in the manufacturer’s Instructions for Use (IFU). The selection of cement viscosity inherently involves a risk–benefit balance: lower viscosity enhances injectability and may promote more homogeneous intravertebral distribution, but it also necessitates vigilant intraoperative monitoring and management to prevent unintended extravasation. Inappropriate application could potentially heighten risks. In this context, our previously established decompressed kyphoplasty technique was applied to reduce intravertebral pressure and guide cement behavior during the injection phase. Near the end of the procedure, posterior cement extravasation was detected under real-time fluoroscopic monitoring while the cement remained in a semi-liquid, unpolymerized state. In response to this unexpected finding, the injection portal was temporarily converted into a suction channel as a case-specific intraoperative maneuver, allowing aspiration of the extravasated cement while preserving intravertebral structural support. The present case highlights a technical approach applied under carefully controlled conditions, which was successfully applied in the present intraoperative setting; however, further clinical studies are required to evaluate its safety, reproducibility, and broader applicability before it can be considered for routine clinical use.

## 5. Conclusions

The suction-assisted intraoperative maneuver technique described herein demonstrates a feasible, case-specific strategy for managing posterior cement leakage during vertebral augmentation. In this 92-year-old patient with a T8 compression fracture, prompt suction of posteriorly extravasated cement prevented spinal canal compromise, preserved neurological function, and obviated the need for secondary open decompression.

While further clinical validation is required, this case highlights the potential of combining low-viscosity cement injection with a venting-to-suction maneuver under carefully monitored intraoperative conditions. Larger-scale studies are necessary to evaluate the safety, reproducibility, and long-term outcomes of this approach across different leakage patterns. Nevertheless, this case suggests that, in selected intraoperative scenarios, this maneuver may offer a promising option for managing cement leakage in elderly and frail patients with vertebral compression fractures.

## Data Availability

The data used to support the finding of this study are available from the corresponding author upon request.

## References

[1] Gao H., Huang J., Wei Q., He C. (2023). Advances in animal models for studying bone fracture healing. Bioengineering.

[2] Albrecht A.P., Kistler-Fischbacher M., De Godoi Rezende Costa Molino C., Armbrecht G., Freystaetter G., Theiler R., Kressig R.W., Da Silva J.A., Rizzoli R., Wanner G.A. (2025). Prevalence and incidence of osteoporotic vertebral fractures in community-dwelling European older adults: An observational analysis of the DO-HEALTH trial. Osteoporos. Int..

[3] Kakoullis L., Sampsonas F., Karamouzos V., Kyriakou G., Parperis K., Papachristodoulou E., Christophi C., Lykouras D., Kalogeropoulou C., Daoussis D. (2022). The impact of osteoporosis and vertebral compression fractures on mortality and association with pulmonary function in COPD: A meta-analysis. Jt. Bone Spine.

[4] Lyons K.J., Majumdar S.R., Ezekowitz J.A. (2011). The unrecognized burden of osteoporosis-related vertebral fractures in patients with heart failure. Circ. Heart Fail..

[5] Alpantaki K., Dohm M., Korovessis P., Hadjipavlou A.G. (2018). Surgical options for osteoporotic vertebral compression fractures complicated with spinal deformity and neurologic deficit. Injury.

[6] Chen S., Yang D., Zhuo C., Zhou Z., Aleem H.B., Huang L., Chen H. (2024). Comparative analysis of percutaneous vertebroplasty and kyphoplasty in the treatment of Stage III Kummell’s disease without neurological symptoms: A retrospective study. J. Orthop. Surg. Res..

[7] Deng R.-Y., Zhan P. (2025). Clinical Effectiveness of Percutaneous Vertebroplasty in Elderly Patients with Osteoporotic Lumbar Vertebral Fractures: A Retrospective Study. Curr. Probl. Surg..

[8] Alvi M.A., Zreik J., Yolcu Y.U., Goyal A., Kim D.K., Kallmes D.F., Freedman B.A., Bydon M. (2020). Comparison of costs and postoperative outcomes between vertebroplasty and kyphoplasty for osteoporotic vertebral compression fractures: Analysis from a state-level outpatient database. World Neurosurg..

[9] Encalada S., Hunt C., Duszynski B., Salmasi V., Scholten P., Zhao Z., Rappard G., Rivers W.E., Vu T.-N., Lobel S. (2025). The effectiveness of balloon kyphoplasty compared to conservative treatment for osteoporotic vertebral compression fractures: A systematic review and meta-analysis. Interv. Pain Med..

[10] Daher M., Kreichati G., Kharrat K., Sebaaly A. (2023). Vertebroplasty versus kyphoplasty in the treatment of osteoporotic vertebral compression fractures: A meta-analysis. World Neurosurg..

[11] You Z., Wu K., Jiang Y., Chen J. (2025). Effect of vertebral kyphoplasty versus vertebroplasty on pain and indicators of imaging parameters of the injured vertebrae in patients with osteoporotic vertebral compression fractures: A meta-analysis. J. Orthop. Surg. Res..

[12] Cerny J., Soukup J., Petrosian K., Loukotova L., Novotny T. (2024). Efficacy and complication rates of percutaneous vertebroplasty and kyphoplasty in the treatment of vertebral compression fractures: A retrospective analysis of 280 patients. J. Clin. Med..

[13] Wu Y., Zhou Z., Lu G., Ye L., Lao A., Ouyang S., Song Z., Zhang Z. (2025). Risk factors for cement leakage after percutaneous vertebral augmentation for osteoporotic vertebral compression fractures: A meta-analysis. Int. J. Surg..

[14] Sheen S., Hasan P., Sun X., Wang J., Tatsui C., Nouri K., Javed S. (2025). Retrospective Analysis of Cement Extravasation Rates in Vertebroplasty, Kyphoplasty, and Bone Tumor Radiofrequency Ablation. J. Clin. Med..

[15] Yang F., Liu Z., Li P., Zhu Q., He Q., Liang Y., Zhang B. (2023). Analysis of Potential Risk Factors for Cement Leakage into Paraspinal Veins after Vertebroplasty for Acute Osteoporotic Vertebral Fractures Based on a 3D Reconstruction Technique: A Retrospective Matched Case–Control Study. Orthop. Surg..

[16] Baek I.-H., Park H.-Y., Kim K.-W., Jang T.-Y., Lee J.-S. (2021). Paraplegia due to intradural cement leakage after vertebroplasty: A case report and literature review. BMC Musculoskelet. Disord..

[17] Mao Z., Xiong Z.-H., Li J.-F. (2024). Thoracic spinal cord injury and paraplegia caused by intradural cement leakage after percutaneous kyphoplasty: A case report. World J. Clin. Cases.

[18] Cui Y., Pan Y., Lin Y., Mi C., Wang B., Shi X. (2022). Risk factors for predicting cement leakage in percutaneous vertebroplasty for spinal metastases. J. Orthop. Sci..

[19] Coniglio A., Rava A., Fusini F., Colò G., Massè A., Girardo M. (2021). Effectiveness and reliability of cannulated fenestrated screws augmented with polymethylmethacrylate cement in the surgical treatment of osteoporotic vertebral fractures. J. Craniovertebral Junction Spine.

[20] Chu W., Tsuei Y.-C., Liao P.-H., Lin J.-H., Chou W.-H., Chu W.-C., Young S.-T. (2013). Decompressed percutaneous vertebroplasty: A secured bone cement delivery procedure for vertebral augmentation in osteoporotic compression fractures. Injury.

[21] Cheng S.-H., Chou W.-H., Tsuei Y.-C., Chu W., Chu W.-C. (2024). Assessment of cement leakage in decompressed percutaneous kyphoplasty. J. Clin. Med..

[22] Lu H.-T., Lin J.-Y., Tsuei Y.-C., Hsu Y.-F., Chen C.-Y., Cheng S.-H., Chu W., Li C., Chu W.-C. (2023). Impact of aspiration percutaneous vertebroplasty in reducing bone cement leakage and enhancing Distribution—An ex vivo study in goat vertebrae. Bioengineering.

[23] Lan Y., Chen S., Lan G., Li C., Wei J. (2025). Global, regional, and national burden of fracture of vertebral column, 1990–2021: Analysis of data from the global burden of disease study 2021. Front. Public Health.

[24] Ciatti C., Asti C., Maniscalco P., Rinaldi M., Pirellas G., Caggiari G., Pisanu F., Sanna A., Doria C. (2025). The Use of Polymethylmethacrylate Cement in Percutaneous Vertebroplasty Versus Conservative Management: How to Treat Osteoporotic Vertebral Compression Fractures. Medicina.

[25] Zhang T.-Y., Zhang P.-X., Xue F., Zhang D.-Y., Jiang B.-G. (2020). Risk factors for cement leakage and nomogram for predicting the intradiscal cement leakage after the vertebra augmented surgery. BMC Musculoskelet. Disord..

[26] Hou J.-G., Zhang N., Chen G.-D. (2023). Factors affecting cement leakage in percutaneous vertebroplasty: A retrospective cohort study of 309 patients. Eur. Rev. Med. Pharmacol. Sci..

[27] Mao Y., Wu W., Zhang J., Ye Z. (2023). Prediction model of adjacent vertebral compression fractures after percutaneous kyphoplasty: A retrospective study. BMJ Open.

[28] Tang B., Xu S., Chen X., Cui L., Wang Y., Yan X., Liu Y. (2021). The impact of intravertebral cleft on cement leakage in percutaneous vertebroplasty for osteoporotic vertebral compression fractures: A case-control study. BMC Musculoskelet. Disord..

[29] Tang B., Cui L., Chen X., Liu Y. (2021). Risk factors for cement leakage in percutaneous vertebroplasty for osteoporotic vertebral compression fractures: An analysis of 1456 vertebrae augmented by low-viscosity bone cement. Spine.

[30] Tan L., Wen B., Guo Z., Chen Z. (2020). The effect of bone cement distribution on the outcome of percutaneous Vertebroplasty: A case cohort study. BMC Musculoskelet. Disord..

[31] Zhou C., Liao Y., Huang S., Li H., Zhu Z., Zheng L., Wang B., Wang Y. (2022). Effect of cement distribution type on clinical outcome after percutaneous vertebroplasty for osteoporotic vertebral compression fractures in the aging population. Front. Surg..

[32] Yang K., Zhu X., Sun X., Shi H., Sun L., Ding H. (2025). Bone cement distribution patterns in vertebral augmentation for osteoporotic vertebral compression fractures: A systematic review. J. Orthop. Surg. Res..

[33] Luo A.-J., Liao J.-C., Chen L.-H., Lai P.-L. (2022). High viscosity bone cement vertebroplasty versus low viscosity bone cement vertebroplasty in the treatment of mid-high thoracic vertebral compression fractures. Spine J..

[34] Zhan Y., Bao C., Yang H., Li L., Yan L., Kong L., Hao D., Wang B. (2023). Biomechanical analysis of a novel bone cement bridging screw system combined with percutaneous vertebroplasty for treating Kummell’s disease. Front. Bioeng. Biotechnol..

[35] Gao Y., Zheng J., Yao K., Wang W., Tan G., Xin J., Li N., Chen Y. (2024). Construction of a nomogram to predict the probability of new vertebral compression fractures after vertebral augmentation of osteoporotic vertebral compression fractures: A retrospective study. Front. Med..

[36] Zhang Z.-F., Liu D.-H., Wu P.-Y., Xie C.-L., Qin F.-W., Huang H. (2018). Ultra-early injection of low-viscosity cement in vertebroplasty procedure for treating osteoporotic vertebral compression fractures: A retrospective cohort study. Int. J. Surg..

[37] Wang Q., Sun C., Zhang L., Wang L., Ji Q., Min N., Yin Z. (2022). High-versus low-viscosity cement vertebroplasty and kyphoplasty for osteoporotic vertebral compression fracture: A meta-analysis. Eur. Spine J..

[38] Viola R., Aslan S., Al-Smadi M.W., Gati A., Szilágyi K., Foglar V., Viola Á. (2025). Effect of Cold Saline Pre-Washing on Cement Leakage in Vertebroplasty: A Novel Approach. J. Clin. Med..

[39] Kochai A., Enercan M., Kahraman S., Ozturk C., Hamzaoglu A. (2018). The effect of mechanical aspiration of the vertebral body on pulmonary arterial pressure before cement injection in the vertebroplasty procedure. J. Orthop. Surg..

[40] Wu S.C.-H., Luo A.-J., Liao J.-C. (2022). Cement augmentation for treatment of high to mid-thoracic osteoporotic compression fractures, high-viscosity cement percutaneous vertebroplasty versus balloon kyphoplasty. Sci. Rep..

[41] Li D., Tao L., Su Q., Zhang X., Wu X. (2025). Warning line for preventing bone cement leakage in surgery involving percutaneous kyphoplasty for osteoporotic vertebral compression fractures. Front. Surg..

